# Combined Chibby and β-Catenin Predicts Clinical Outcomes in Patients with Hepatocellular Carcinoma

**DOI:** 10.3390/ijms21062060

**Published:** 2020-03-17

**Authors:** Ming-Chao Tsai, Chao-Cheng Huang, Yu-Ching Wei, Ting-Ting Liu, Ming-Tsung Lin, Li-Na Yi, Pey-Ru Lin, Chih-Chi Wang, Tian-Huei Chu, Chang-Chun Hsiao, Tsung-Hui Hu, Ming-Hong Tai

**Affiliations:** 1 Division of Hepato-Gastroenterology, Department of Internal Medicine, Kaohsiung Chang Gung Memorial Hospital and Chang Gung University College of Medicine, Kaohsiung 83301, Taiwan; tony0779@gmail.com (M.-C.T.); dr.lin520@gmail.com (M.-T.L.); yih10062002@yahoo.com.tw (L.-N.Y.); peyrulin@hotmail.com (P.-R.L.); 2 Graduate Institute of Clinical Medical Sciences, Chang Gung University College of Medicine, Taoyuan City 33302, Taiwan; 3 Department of Pathology, Kaohsiung Chang Gung Memorial Hospital, Chang Gung University College of Medicine, Kaohsiung 83301, Taiwan; huangcc@cgmh.org.tw (C.-C.H.); liutt107@cgmh.org.tw (T.-T.L.); 4 Department of Pathology, Kaohsiung Municipal Ta-Tung Hospital, Kaohsiung Medical University Hospital, Kaohsiung Medical University, Kaohsiung 80145, Taiwan; yobeary@gmail.com; 5 Department of Medical Laboratory Science, I-Shou University, Kaohsiung 84001, Taiwan; 6 Department of Surgery, Kaohsiung Chang Gung Memorial Hospital and Chang Gung University College of Medicine, Kaohsiung 83301, Taiwan; ufel4996@gmail.com; 7 Biobank and Tissue Bank, Kaohsiung Chang Gung Memorial Hospital, Kaohsiung 83301, Taiwan; skbboyz0817@gmail.com; 8 Graduate Institute of Clinical Medical Sciences, College of Medicine, Chang Gung University, Division of Pulmonary and Critical Care Medicine, Kaohsiung Chang Gung Memorial Hospital, Kaohsiung 83301, Taiwan; cchsiao@mail.cgu.edu.tw; 9 Institute of Biomedical Sciences, National Sun Yat-Sen University, Kaohsiung 80424, Taiwan

**Keywords:** Chibby, hepatocellular carcinoma, β-catenin

## Abstract

Chibby is an antagonist of β-catenin and is considered a potential tumor suppressor protein, but the role of Chibby in hepatocellular carcinoma (HCC) has not been characterized. The expression patterns of Chibby and β-catenin in HCC specimens and paired adjacent noncancerous tissues were measured by Western blotting and immunohistochemistry. The correlations between Chibby expression and clinicopathological parameters were analyzed. Then the biological functions of Chibby were analyzed in vitro. The Chibby protein was significantly downexpressed in human primary HCC tissues compared to that in matched adjacent normal liver tissue and is a risk factor for HCC recurrence and shorter survival. Furthermore, we found that in HCC tissues the high expression of β-catenin with low expression of Chibby in the nuclei was an independent predictor for disease-free survival (DFS) (*p* = 0.012) and overall survival (OS) (*p* = 0.005). Subsequent genetic manipulation in vitro studies revealed that Chibby knockdown induced the expression of β-catenin and C-myc, cyclin D1 protein, which promoted cell proliferation and invasiveness. In contrast, overexpression of Chibby decreased β-catenin expression and inhibited the cell proliferation and invasiveness. Our results suggest that low expression of Chibby was associated with advanced tumor-node-metastasis (TNM) stage and poor differentiation. Furthermore, the combination of Chibby and β-catenin can predict poor prognosis in patients with HCC. Chibby inhibited HCC progression by blocking β-catenin signaling in vitro. Chibby is a biomarker and may be a potential therapeutic target for HCC.

## 1. Introduction

Hepatocellular carcinoma (HCC) is the second cause of cancer-related mortality worldwide, accounting for an estimated 600,000 deaths annually [1]. Although much is known about the cellular changes that lead to HCC and the etiologic agents responsible for most cases of HCC (i.e., hepatitis B and C viral infections and alcohol abuse), the molecular pathogenesis of HCC is not well understood [2,3]. A study clarifying the underlying biology of HCC development at the molecular level would improve the application of currently available treatment modalities and offer new potential treatment strategies.

Aberrant activation of Wnt/β-catenin signaling has been shown to play a major role in the pathogenesis of HCC over the last two decades [4,5]. Mutations of *CTNNB1* (the β-catenin gene) have been discovered in around 20%–40% of all HCC cases, and define *CTNNB1* as the most frequently mutated gene in HCCs [6,7]. There is a strong association between nuclear accumulation of β-catenin and its mutations. Previous studies have found an association between poor prognosis in HCC patients with nuclear β-catenin accumulation in high-grade HCC tumors, suggesting that β-catenin promotes tumor proliferation and progression [8,9]. According to these observations, interfering with the Wnt/β-catenin signaling might be a potential target for HCC therapy.

Chibby is a 15-kDa, highly conserved protein that was originally identified as a β-catenin antagonist using the C-terminal transactivation domain of β-catenin in 2003 [10]. Chibby physically interacts with the C-terminal domain of β-catenin and competes with Tcf/Lef transcription factors, leading to repression of Wnt target genes. Initial studies indicated RNAi knockdown of Chibby results in hyperactivation of this signaling pathway [10,11]. As we know, the Wnt/β-catenin pathway is frequently activated in HCC through *CTNNB1* mutations that activate β-catenin [5]. Theoretically, Chibby, a Wnt/β-catenin antagonist, should have a potential effect in HCC. However, the impact and interaction between β-catenin and Chibby in the tumorigenesis of HCC have not been well investigated.

In the present study, the interaction between Chibby and β-catenin was investigated in HCC tissues and its clinical significance in HCC patients, and the role of Chibby in HCC proliferation and invasion by gene regulation to clarify its clinical significance was also explored.

## 2. Results

### 2.1. Low Expression of Chibby Correlates with High Stage of HCC

**Figure 1:**
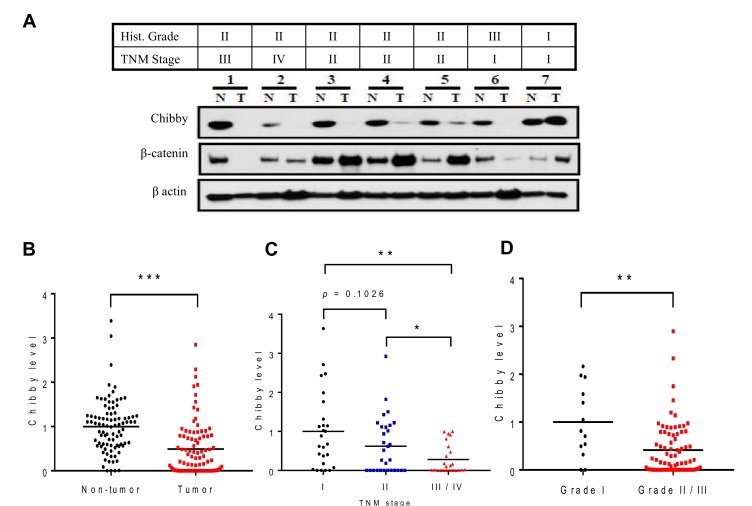
Chibby is downexpressed in hepatocellular carcinoma (HCC) and correlated with advanced stage. (**A**) The Western blotting (WB) analyses of Chibby and β-catenin protein expression in seven pairs of HCC tissues (T) and their paired non-tumor tissues (N). Patient 1 was histology grade I and TNM stage III; patient 2 was histology grade II and TNM stage IV; patient 3-5 were histology grade II and TNM stage II; patient 6 was histology grade III and TNM stage I; patient 7 was histology grade I and TNM stage I. β-actin was used as a loading control. Chibby protein levels were significantly lower in HCC tumors (**B**), high TNM stage (**C**), and high histology grade (**D**). * *p* < 0.05; ** *p* < 0.01; *** *p* < 0.005.

To investigate whether Chibby is dysregulated in human HCC, we performed Western blotting on 90 pairs of HCC patients (Figure 1A). Compared with the paired non-tumor tissues, the Chibby protein expression was significantly downregulated in tumor tissues (Figure 1B), in cases of high tumor-node-metastasis (TNM) stage (Figure 1C), and in cases of high histology grade (Figure 1D). The findings suggested that reduced Chibby expression was associated with advanced HCC. However, there was no significant association between Chibby and β-catenin protein expression both in HCC tissues and their paired non-tumor tissues.

**Figure 2:**
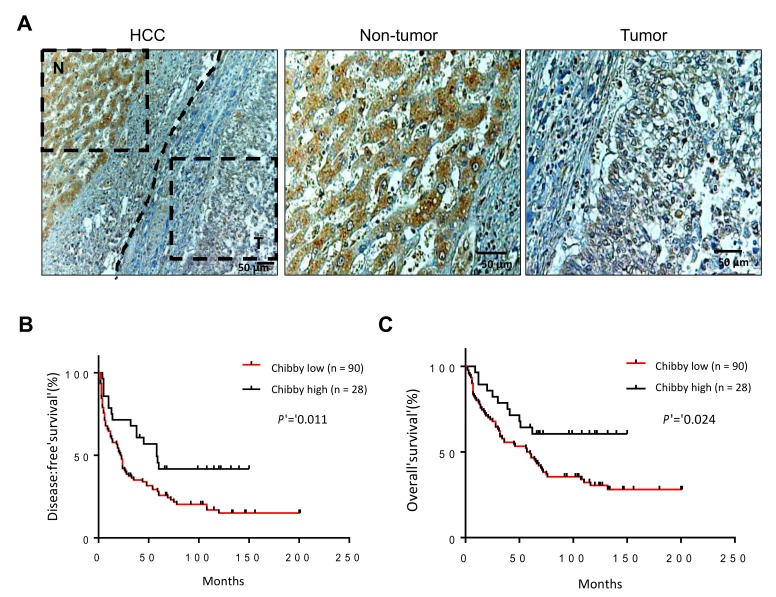
Chibby is downexpressed in HCC patients and associated with poor prognosis by immunohistochemistry (IHC) analysis. (**A**) IHC of Chibby in HCC tissue specimens (T) and respective non-tumor tissue specimens (N). Samples were classified into two groups according to Chibby expression: Chibby high group (28 of the 118 (23.7%)) and Chibby low (90 of the 118 (76.3%)). Kaplan–Meier disease-free survival (**B**) and overall survival curves (**C**) of “Chibby high” and “Chibby low” group HCC patients. Scale bars: 50 μm.

To identify the profile of Chibby in HCC, immunohistochemistry (IHC) staining was performed to detect Chibby protein expression in 156 paraffin-embedded HCC specimens. Chibby immunostaining was detected in both tumor and non-tumor cells (Figure 2A). All specimens were divided into high and low expression groups according to the mean immunohistochemistry scores (described in the immunohistochemical staining and scoring section). Of them, 28 (17.9%) patients were assigned to the high Chibby expression group and 90 (57.7%) patients to the low Chibby expression group. The other 38 (24.4%) patients were undetermined. The survival analysis indicated that patients with high Chibby expression had a significant disease-free survival (DFS) (*p* = 0.011, Figure 2B) and overall survival (OS) (*p* = 0.024, Figure 2C) than those with low expression Chibby level.

We also examined the expression of Chibby in tumor parts and observed subcellular localization of Chibby staining in both cytoplasm and nuclei. Of them, 122 (78.2%) were detected with Chibby in the cytoplasm and 72 (46.2%) in the nuclei.

However, both showed no significant differences in DFS and OS (Table S1).

### 2.2. Correlation between Chibby Expression and β-Catenin Expression in HCC Specimens

**Figure 3:**
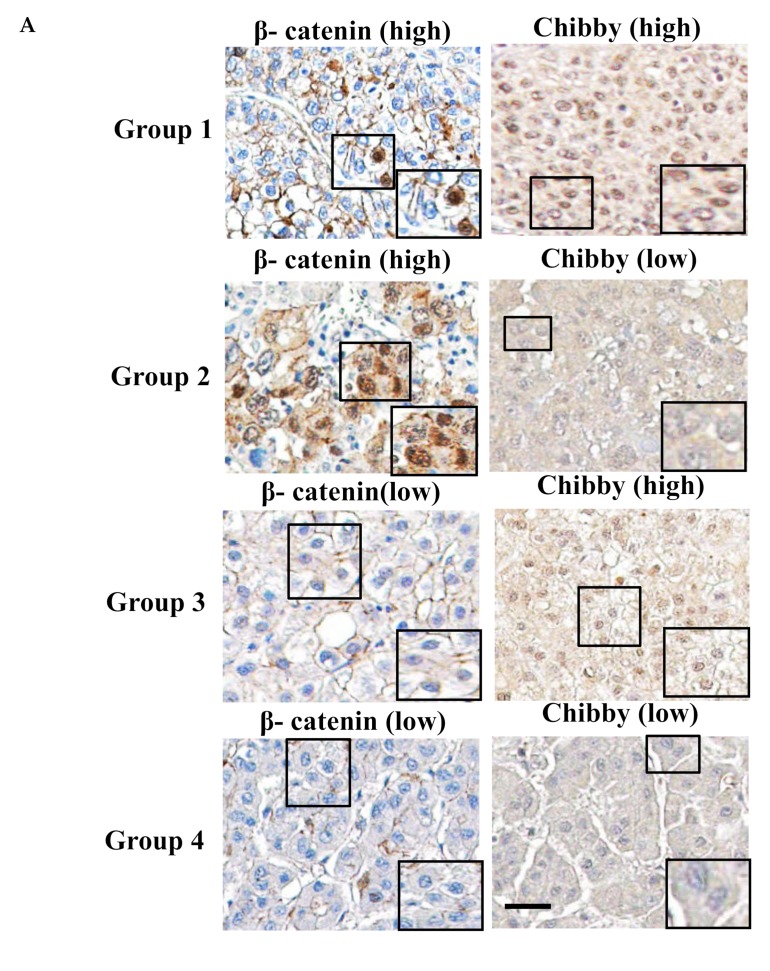

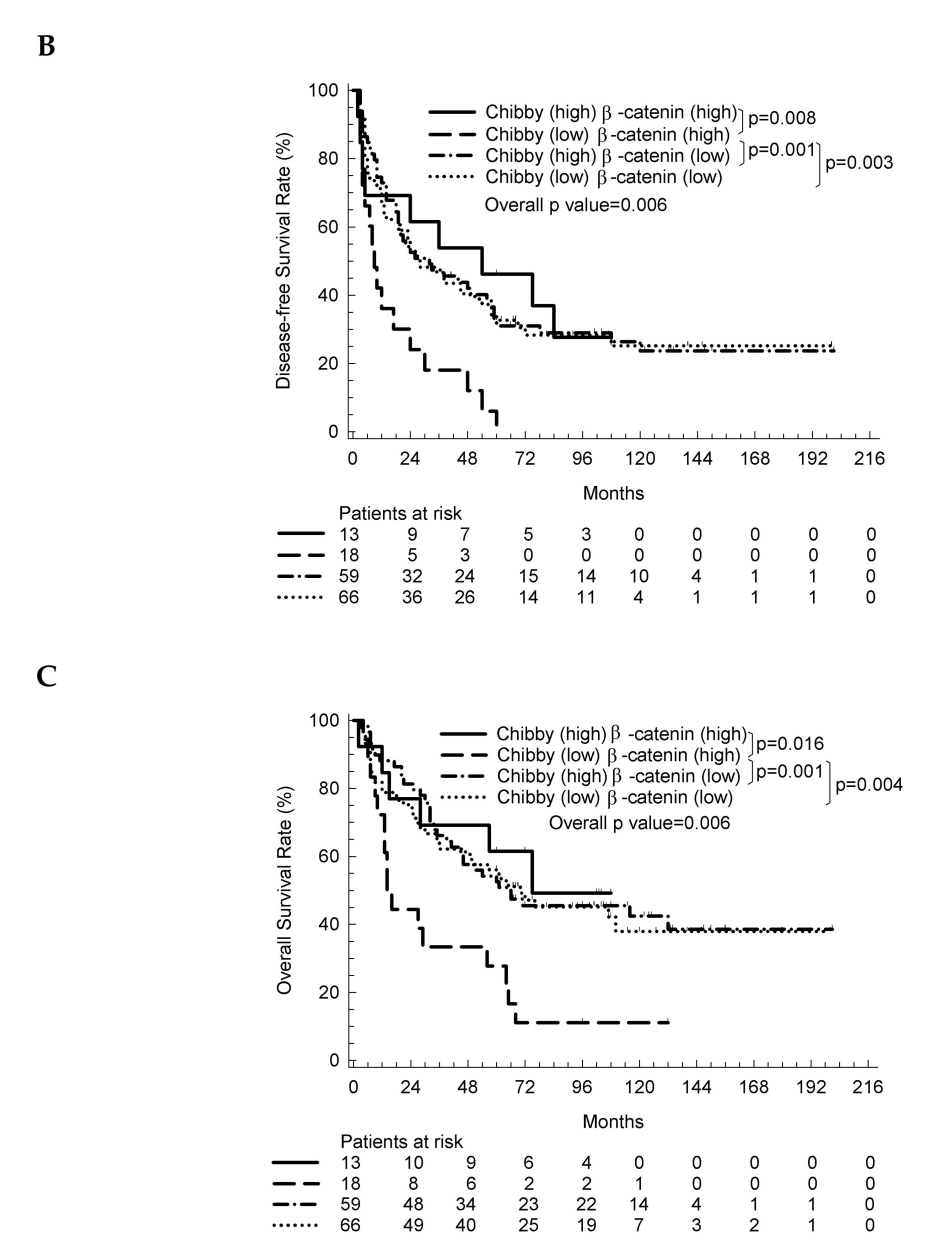
The high expression of nuclear β-catenin and low expression of nuclear Chibby in HCC tissue indicates poor disease-free survival and overall survival. (**A**) There are four nuclear expression types of β-catenin and Chibby in representative HCC cases which were examined by immunohistochemistry (original magnification, 400×), Scale bar, 100 μm: Group 1: β-catenin (high)/Chibby (high), β-catenin and Chibby were expressed highly in cell nuclei concurrently; Group 2: β-catenin (high)/Chibby (low), β-catenin was highly expressed in cell nuclei, but Chibby was low expressed in cell nuclei; Group 3: β-catenin (low)/Chibby (high), β-catenin was low expressed in cell nuclei, but Chibby was highly expressed in cell nuclei; Group 4: β-catenin (low)/Chibby (low), concurrently, β-catenin and Chibby were low expressed in cell nuclei. “High” was defined as the average score of β-catenin- or Chibby-expressed unclear in five fields (400× magnification) higher than the mean score of all samples; “low” was defined as the score lower than the mean score of all samples. Patients with high expression of nuclear β-catenin and low expression of nuclear Chibby displayed poor disease-free survival (B) and overall survival (C).

To further investigate the correlation between Chibby and β-catenin and HCC characteristics, we examined β-catenin status by IHC staining. There were three types of β-catenin in HCC tissues: nuclei (*n* = 31, 19.9%), cytoplasm (42, 26.9%), and membrane types (96, 61.5%) (Figure S1). Statistical analysis demonstrated that only HCC with nuclear β-catenin had a significantly shorter OS (*p* = 0.048) (Table S1). To further explore the association between Chibby and β-catenin in tumors, we further scored the intratumoral nuclear expression of Chibby and β-catenin as high or low according to the mean number of positive nuclear staining by IHC staining (see Material and Methods). All patients were divided into four groups according to the results of the IHC staining of Chibby and β-catenin nuclear expression (Figure 3A): high expression of β-catenin and Chibby [β-catenin (high)/Chibby (high), *n* = 13] (Figure 3A, group 1); high expression of β-catenin but low expression of Chibby [β-catenin (high)/Chibby (low), *n* = 18] (Figure 3A, group 2); low expression of β-catenin but high expression of Chibby [β-catenin (low)/Chibby (high), *n* = 59] (Figure 3A, group 3), and low expression of β-catenin and Chibby [β-catenin (low)/Chibby (low), *n* = 66] (Figure 3A, group 4). We further examined the correlation between the nuclear expression of β-catenin and Chibby and clinicopathologic parameters (Table 1). The results indicated that only tumor number (multiple vs. single) was significantly correlated with the nuclear expression of β-catenin and Chibby (*p* = 0.025). Of them, patients with β-catenin (high)/Chibby (low) had the highest proportion of multiple HCC (50%) than the other three groups.

### 2.3. The Nuclear Expression of Chibby Predict Disease-Free Survival and Overall Survival in HCC Patients with Nuclear Expression of β-Catenin

All HCC patients were divided into four groups according to the results of the IHC staining of Chibby and β-catenin unclear expression, as mentioned above. We found that patients with high expression of nuclear β-catenin and low expression of nuclear Chibby have a significantly poor DFS and OS compared with others (*p* = 0.006, both) (Figure 3B,C). To evaluate the potential of using both Chibby and β-catenin nuclear expression in HCC specimens for the prognosis of patients with HCC after resection, univariate analysis showed that alpha-fetoprotein (AFP) level > 200 ng/mL, liver cirrhosis, tumor size > 5 cm, multiple tumor number, TNM stage III and IV, poor histology grade, and HCC with β-catenin (high) and Chibby (low) were risk factors for recurrent HCC. Multivariate analysis showed AFP level > 200 ng/mL (*p* = 0.001), liver cirrhosis (*p* = 0.004), TNM stage III and IV (*p* < 0.001), and HCC with β-catenin (high) and Chibby (low) (*p* = 0.012) were significantly associated with recurrent HCC (Table 2). In overall survival analysis, univariate analysis showed that AFP level > 200 ng/mL, tumor size > 5 cm, TNM stage III and IV, poor histology grade, and HCC with β-catenin (high) and Chibby (low) were risk factors for mortality; multivariate analysis showed AFP level > 200 ng/mL (*p* = 0.021), TNM stage III and IV (*p* < 0.001), and β-catenin (high) and Chibby (low) (*p* = 0.005) were significantly associated with mortality (Table 3).

### 2.4. Knockdown of Chibby Promotes β-Catenin Signaling and HCC Cell Proliferation and Invasion

**Figure 4:**
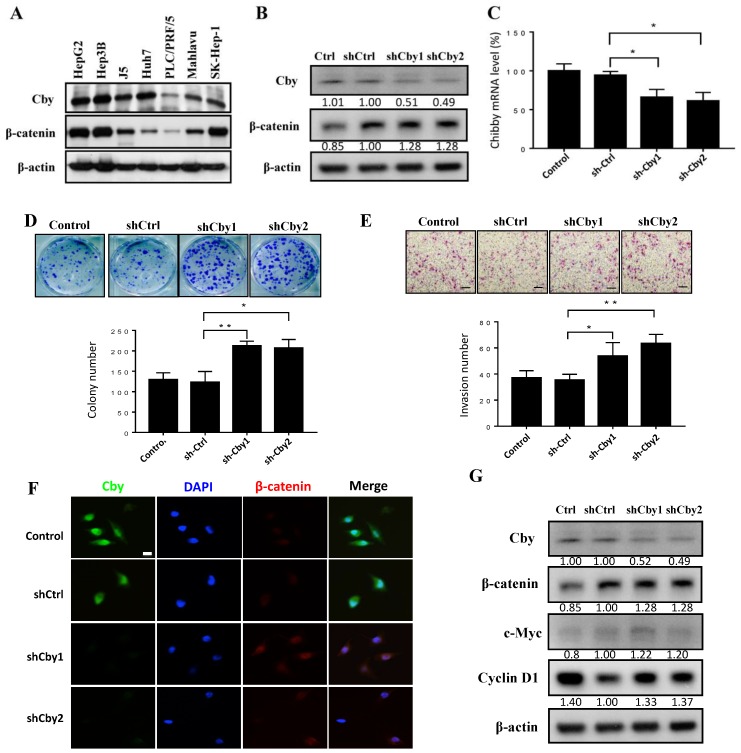
Knockdown of Chibby expression increases cell proliferation and invasion of HCC cells. (**A**) Western blot analysis of Chibby protein in a variety of HCC cell lines. Two plasmids expressing *Chibby*-specific shRNA sequences were transfected into Huh7 cells, and the plasmid sh-control was designed as a scramble control. The protein level of Chibby was analyzed by Western blot (**B**), and the RNA level was detected by qRT-PCR (**C**). The Huh7 cells transfected with shChibby promoted cell proliferation and invasiveness by colon-formation assay (**D**) and Boyden chamber system (**E**). (**F**) Immunofluorescence was used to detect the upregulation of β-catenin in Huh7 cells after transfection with shChibby. In cells treated with shChibby, prominent β-catenin staining was detected. (**G**) Chibby knockdown activates the Wnt/β-catenin pathway in Huh7 cells. Analysis of β-catenin, C-Myc, and cyclin D1 expression by Western blot in Huh 7 cells treated with shChibby. β-actin was used as the loading control. Data represent mean ± SE from three independent analyses. Scale bar, 100 μm, * *p* < 0.05 and ** *p* < 0.01 vs. shControl.

Based on the above observations, we speculated that Chibby has an antioncogenic function on HCC. First, we primarily examined seven HCC cell lines (HepG2, Hep3B, J5, Huh 7, PLC, Mahlavu, and SK-Hep-1) to assess whether HCC cell lines endogenously express Chibby by Western blot. We found that higher expression of Chibby in HepG2, Hep3B, J5, and Huh7, compared with PLC, Mahlavu, and SK-Hep-1, which showed relatively lower expression of Chibby (Figure 4A). Then we chose two cell lines, Huh7 and SK-Hep-1, for further in vitro study. To investigate the effect of Chibby silence on the HCCs, we knocked down Chibby by shRNA, which specifically targets Chibby in the Huh7 cell line at both mRNA and protein levels as compared to that in the scramble (Figure 4B and 4C), which resulted in significantly increased colony formation (*p* < 0.05) (Figure 4D) and cell invasive number (*p* < 0.05) (Figure 4E). Moreover, the inverse protein expression pattern between Chibby and β-catenin was observed. The immunofluorescence results showed that when Huh7 cells were knocked down by transfection Chibby-siRNA, the expression of β-catenin enhanced mainly in the nuclei (Figure 4F). By Western blot analysis, silencing the expression of Chibby increased the expression of β-catenin, as well as c-myc and cyclin D1 (Figure 4G), the two known downstream target genes of the β-catenin/Tcf complex, and play crucial roles in proliferation [12,13]. These results provide evidence that Chibby inhibits HCC cell proliferation.

### 2.5. Expression of Chibby Inhibits β-Catenin Signaling and HCC Cell Proliferation and Invasion

**Figure 5:**
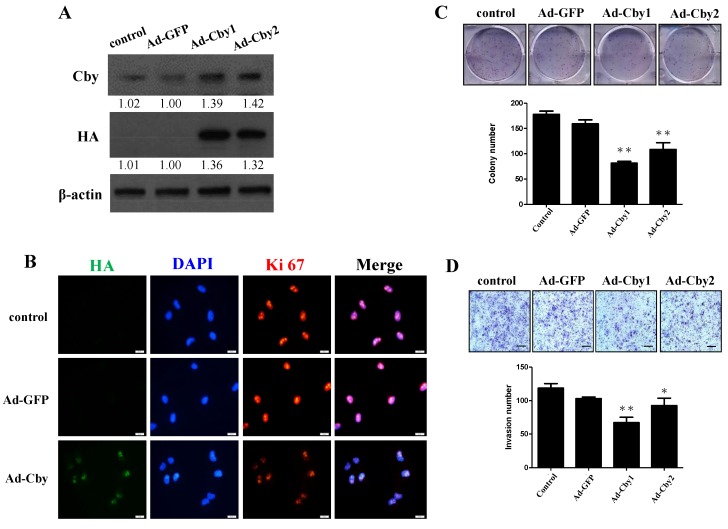
Overexpression of Chibby expression inhibits cell proliferation and invasion of HCC cells. (**A**) Adenovirus-mediated ectopic expression of Chibby and enhanced the expression of Chibby in Huh7 cells. After 48 h of infection with adenoviral vectors (Ad-GFP or Ad-Chibby) at different multiplicity of infection, the protein lysates from the Huh7 cells were harvested to determine the ectopic gene expression using Western blot analysis. Ad-GFP was designed as the control vector. (**B**) Representative immunofluorescent images of Ki67 in Huh7-normal control, Huh7-Ad-GFP, and Huh7-Ad-Chibby cells. (**C**,**D**) The enhancement of Chibby in Huh7 cells transfected with Ad-Chibby suppressed cell proliferation and invasiveness by colon-formation assay and the Boyden chamber system, respectively. Data represent mean ± SE from three independent analyses. Scale bar, 100 μm, * *p* < 0.05 and ** *p* < 0.01 vs. Ad-GFP group.

To further verify the role of Chibby, we overexpressed Chibby in Huh7 cells with an adenovirus carrying Chibby (wild type). The overexpression of the Chibby proteins was confirmed by Western blot analysis (Figure 5A). As shown in Figure 5B, overexpression Chibby decreased the expression of Ki67 by immunofluorescence. In the functional assay, overexpression of Chibby significantly suppressed cell proliferation and invasiveness (Figure 5C,D). When silencing the expression of Chibby in SK-Hep-1 cell, the β-catenin, c-myc, and cyclin D1 protein were increasing (Figure S3A), accompanying β-catenin enhancement by immunofluorescence (Figure S3B). Chibby downexpression also increased SK-Hep-1 cell invasiveness and colony formation (Figure S3C,D). Chibby overexpression had the opposite effect on SK-Hep-1 cells (Figure S4). Taken together, these data demonstrated that Chibby is critical for Wnt/β-catenin signaling-induced HCC cell proliferation and invasion.

## 3. Discussion

In the present study, we first provided evidence that Chibby is downexpressed in HCC samples and correlated with advanced TNM stage, histology grade, postoperative recurrence, and poor survival of patients. The findings suggest that Chibby plays an antioncogenic role in HCC and may constitute a prognostic factor for patients with HCC after resection. Furthermore, we observed that HCC patients with high expression of β-catenin and low expression of Chibby in tumor nuclei had poor outcomes in HCC recurrence and overall survival. To our knowledge, this is the first study using the expression of Chibby to predict outcomes in HCC patients after resection.

The Wnt/β-catenin signaling pathway is aberrantly activated in a wide range of HCC patients. Nuclear accumulation of β-catenin is associated with poor prognosis in HCC patients, suggesting that β-catenin promotes tumor proliferation and progression [8,9]. The end-point of activation of the Wnt/β-catenin signaling is the formation of β-catenin/Lef complexes in the nuclei that lead to the constitutive activation of a genetic program promoting cancer development. Hence, drugs designed to stabilize β-catenin translocate to the nucleus and disrupt the Lef binding to β-catenin appear to be a feasible approach for inhibiting cancer development induced by aberrant activation of the Wnt/β-catenin pathway. Chibby is a newly found β-catenin binding partner, which represses β-catenin-mediated transcriptional activation by competing with Lef factors for β-catenin binding [10,14]. However, a potential tumor suppressor function of Chibby remains poorly defined in HCC.

The present results are consistent with other studies, which documented that overexpression of Chibby is correlated with less aggressive cancer phenotypes in many cancers, such as colorectal cancer, lung cancer, and laryngeal squamous cell carcinoma [15,16,17]. However, the association between the expression level of Chibby and clinical outcomes has not been reported previously. Our study is the first to indicate an association between the expression of Chibby and the clinical outcomes in HCC patients after resection. Chibby expression is a prognostic factor for DFS and OS in HCC patients, indicating that it has potential as a biomarker for prognostic prediction in HCC. Thus, it could help to identify more aggressive subtypes of cancer in patients who need to undergo postoperative adjuvant target therapy.

It is worth noting that there is no significance between HCC patients with high and low expression of nuclear Chibby. We further explored the association between β-catenin and Chibby in HCC. The results indicated that high expression of β-catenin and low expression of Chibby in the nuclei in HCC tissues is an independent risk factor for HCC recurrence and overall survival. Taken together, Chibby plays an antioncogenic role in HCC patients with the expression of nuclear β-catenin, especially. However, there are no associations between the expression of Chibby in tumor tissue and gender, age, clinical staging, and tumor differentiated degree in our clinical observations. The factors regulating the expression of Chibby in HCC are still questionable.

We manipulated the expression of Chibby in Huh 7 HCC cell lines to see whether Chibby regulates the biological behavior of HCC by targeting the Wnt/β-catenin signaling activity. Our results in vitro were consistent with our clinical findings, which showed that enforced Chibby expression resulted in the attenuation of HCC cell proliferation and invasiveness, while Chibby knockdown promoted cell proliferation and invasiveness. Meanwhile, in these cells with overexpression or knockdown of Chibby, β-catenin is predominantly inversely expressed, and its signaling activity is affected consequently. Xu et al. observed the enforced expression of Chibby effectively suppressed laryngeal carcinoma cell growth by inhibiting Wnt/β-catenin signaling [16]. Fischer et al. also found that Chibby negatively modulates endogenous β-catenin signaling in colon cancer cells and suppresses cell growth [15]. Our results are consistent with prior studies in different cancers. Therefore, our in vitro experiments indicated a strong inverse correlation between Chibby and β-catenin levels and provided a novel means for the therapeutic intervention of Wnt/β-catenin-driven tumors.

In addition to the Huh7 HCC cell line, we also regulated the Chibby expression into the SK-Hep-1, a liver adenocarcinoma cell line, and the results are consistent with the HCC cell lines. Taking all together, we suggested that Chibby can regulate different types of liver tumors, such as HCC and adenocarcinoma. Further clinical studies that enroll different liver tumors, such as cholangiocarcinoma or liver metastatic tumor, need to be further clarified.

Although prior studies have documented that overexpression of Chibby in colorectal, lung, and laryngeal squamous cell carcinoma correlate with less aggressive cancer phenotypes [15,16,17], which are consistent with our results, it is noteworthy that the present study is the first study indicating the association between the expression of Chibby and the clinical outcomes, which is different from the prior studies which were only observation from tumor cell lines. By clinical observation, we found only in the subgroup of patients with high nuclear β-catenin expression, Chibby plays a rescue role to improve the outcomes. In contrast, in HCC patients with low nuclear β-catenin expression, there is no significant association between the expression of Chibby and β-catenin. As we know, HCC has wide variations in etiology and inter- and intra-phenotypes, which are involved by various signal transduction pathways, such as TERT, Wnt/β-catenin, p53, chromatin remodeling complexes and epigenetic regulators, RAS-RAF-MAPK, and the oxidative stress pathway [5]. Therefore, identifying the exact pathway, and then targeting it, is a crucial step in the management of HCC, especially in the era of personalized “precision medicine” [18]. Our findings indicate a good example that Chibby might be useful in the management of HCC with β-catenin expression, but not for all HCC types. Further clinical studies are required to verify this concept in a large cohort.

## 4. Materials and Methods

### 4.1. Patients and Tissue Samples

From August 1990 to August 1999, 156 patients who underwent curative hepatic resection for HCC at the Chang Gung Memorial Hospital, Kaohsiung, Taiwan, were recruited into this study. One hundred and fifty-six HCC paraffin tissues after liver resection were obtained from the pathology department. The diagnosis of HCC was based on the criteria of practice guidelines of the European Association for the Study of the Liver (EASL) or the American Association for the Study of Liver Disease (AASLD) [19,20]. Disease-free survival (DFS) is defined as the duration from the date of operation to the date of recurrence. Overall survival (OS) is counted from the date of surgery to the date of death or December 2015. Survival and mortality were investigated by examining the patients’ final medical records. Clinicopathological parameters, including the demographic data, hepatitis markers, serum AFP levels, tumor numbers, and tumor size, were examined. The clinical stages of HCC patients were established using the American Joint committee on Cancer TNM staging system [21]. Tumor histological grade was followed by the Edmondson–Steiner system [22]. The clinicopathologic characteristics of 156 HCC patients are summarized in Table 4. The median age was 56 years, and 80.1% (*n* = 125) of the patients were male. In addition, there was liver cirrhosis, hepatitis B predominance, and TNM stage I and II predominance. The study was performed in accordance with the current Declaration of Helsinki guidelines. The protocol was approved by the Institutional Review Board of Chang Gung Memorial Hospital, Taiwan (IRB number:201800049B0). Written informed consent was obtained from each patient.

### 4.2. Immunohistochemical Staining and Scoring

The paraffin-embedded tissue blocks were obtained from the department of pathology sectioned for IHC. All slides were incubated overnight at 4 ℃ in a humidified chamber with primary antibodies against Chibby (AP9418b, Abgen, San Diego, CA, USA) and β-catenin (A5441, Sigma, St. Louis, MO, USA). After washing with PBST, the sections were then incubated with peroxidase-conjugated secondary antibodies for 30 min. Finally, antibody staining was visualized with 3,3-diaminobenzidine tetrahydrochloride (DAB; Sigma, St. Louis, MO, USA) in 0.1 M Tris pH 7.2, containing 0.01% H_2_O_2_, and counterstained with Harris’s hematoxylin, and mounted with the dePex medium for microscopic observation.

The intensity of Chibby immunostaining in the tumor and non-tumor parts was evaluated by two independent pathologists using a scoring system. The number of positive cells was divided into five grades (percentage scores): 0 (≤10%), 1 (11%–25%), 2 (26%–50%), 3 (51%–75%), and 4 (>75%). The intensity score was graded from 0 to 3 to represent null (0), weak (1), intermediate (2), and strong (3) intensities (Figure S2). The final score was obtained by multiplying the two scores and ranged from 0 to 12. The ∆score was obtained by the score of HCC minus non-HCC. If ∆score ≤ −2, it was defined as low Chibby expression; conversely, if ∆score ≥ 2, it was defined as high Chibby expression. In addition, we counted the number of positive nuclear stains of Chibby or β-catenin in each one enclosed area. The mean of the five fields was represented as the tumor nuclear Chibby score. The average score of tumors nuclear Chibby in 156 HCC tissues was 15.2 ± 3.7 (mean ± SD) per field under 400× magnification, and this value was designated as the cut-off value for high/low Chibby. Using the same scoring method, the mean score of β-catenin was 46.4 ± 7.9 per field. “High” was defined as the average nuclear stain value (from five independent fields) higher than the cut-off value, and “low” was defined as the value lesser than the cut-off value.

### 4.3. RNA Isolation and Quantitative Reverse-Transcriptase Polymerase Chain Reaction

Total RNA was extracted from the cells using the Quick-RNA™ kit (QIAGEN, 74104, Austin, TX, USA). RNA was quantified using a NanoVue spectrophotometer (GE Healthcare, East Lyme, CT, USA). RNA was reverse-transcribed into cDNA with a PrimeScriptTM RT reagent Kit (Takara #RP037A, Tokyo, Japan). The quantitative real-time PCR was performed with SYBR Premix Ex Taq (Takara; RR041, Tokyo, Japan) and a 7500 fast real-time PCR system (Applied Biosystems, Foster City, CA, USA). Relative mRNA was calculated by the 2^−∆∆CT^ method and normalized to beta-actin. Each PCR reaction was performed in triplicate and averaged. Melting curve analysis was carried out at the end of a 40-cycle amplification to validate the size of the PCR products. The primers were as follows for human Chibby (Forward, TCTTTGGGAATACGTTCAGTCCG; Revers: CCAGGTTCATAGTCGGGGA), and for human β-actin (Forward: CCAACCGCGAGAAGATGA; Reverse: CCAGAGGCGTACAGGGATAG).

### 4.4. Western Blot Analysis

Western blotting was used to detect the expression of Chibby and β-catenin in transfected and non-transfected Chibby HCC cell lines. Cell extracts of cultured HCC cells were prepared by centrifuging the cells in radioimmunoprecipitation assay (RIPA) buffer supplemented with protease inhibitor (Complete Lysis-M kit, Roche Diagnostics, Indianapolis, IN, USA), incubated on ice for 10 min and centrifuged for 10 min at 21,000× *g*. Protein concentration were determined using a Bio-Rad DC kit (Bio-Rad Laboratories, Hercules, CA, USA). Proteins (25–50 µg/lane) were subjected to immunoblot analysis. Blots were developed using the ECL Detection System (Amersham Pharmacia Biotech, Buckinghamshire, England). Western blot membranes were incubated using the following primary antibodies: anti-Chibby (AP9148b, Abgent, San Diego, CA, USA), anti-c-myc (ab32072, Abcam, Cambridge, UK), anti-PCNA (ab92552, Abcam, Cambridge, UK), anti-cyclin D1 (ab134175, Abcam, Cambridge, UK), and anti-β actin (A5441, Sigma, St. Louis, MO, USA). The reactions were detected with a horseradish peroxidase-conjugated secondary antibody (Anti-rabbit and anti-mouse, IgG, Cell Signaling, Danvers, MA, USA). The primary antibodies were incubated overnight at 4 °C, and the secondary antibodies were incubated at 25 °C for 1 h. Densitometric analysis of bands were performed with Image J software (National Institutes of Health; available at http://rsb.info.nih.gov/ij/).

### 4.5. Immunofluorescence Staining

Cells were fixed with 4% paraformaldehyde in PBS at room temperature for 5 min, permeabilized with 0.2% Triton X-100 in PBS for 15 min, rinsed three times with PBS, and blocked with normal goat serum diluted 1:20 or with 5% BSA/PBS for 30 min. Staining was performed with primary rabbit polyclonal Ab to Chibby (AP9148b, Abgent, San Diego, CA, USA), mouse monoclonal Ab to β-catenin (A5441, Sigma, St. Louis, MO, USA), or rabbit polyclonal Ab to Ki67 (NB110-89717, Novus, Littleton, CO, USA) at a dilution of 1:100 at 4 °C overnight. Cells were washed five times with PBS and then incubated with a donkey anti-mouse Texas Red –conjugated secondary antibody for 2 h (Molecular Probe) at room temperature. Finally, the slides were mounted by ProLongR Gold antifade reagent with DAPI (Invitrogen, Eugene, OR, USA) and examined using a Zeiss Axiophot microscope (Zeiss Inc., Boston, MA, USA).

### 4.6. Cell Culture and Transfection Protocols

The Huh7 and SK-Hep1 cell lines provided by Dr. Ming-Hong Tai (National Sun Yat-sen University, Kaohsiung, Taiwan) were maintained in Dulbecco’s modified Eagle’s medium (Hyclone BRL, Rockville, MD, USA) supplemented with 10% fetal calf serum (Hyclone, Denver, CO, USA). Transient transfections were carried out using a lipofectamine 2000 transfection reagent (Invitrogen) for HCC cells. Approximately 1–2 × 10^5^ cells were plated in a 6-well plate and grown in appropriate media to ~50% confluency for transfection. Then each well was treated with a mixture containing 2 μg plasmid DNA and 6 μL lipofectamine2000. The cells were investigated or harvested 24 h after transfection.

### 4.7. Adenoviral Vectors-Mediated Gene Delivery

Adenoviral vectors encoding human Chibby cDNA (GenBank accession number: NM_015373) with N-terminal hemagglutinin tag (Ad-Chibby) and green fluorescent protein (Ad-GFP) were generated through homologous recombination by calcium phosphate protocol. Cells (80%–90% confluence) were infected with adenoviral vectors at different multiplicity of infection (MOI) in a serum-free medium for 1 h. After 1 h adsorption, the virus-containing media were removed, and cells were incubated with fresh complete media.

### 4.8. Knockdown of Endogenous Chibby

Cells were transfected with Chibby shRNA plasmid (sc-72890, Santa Cruz, Dallas, TX, USA), a pool of three Chibby-specific plasmids, each encoding 19–25 nucleotide long shRNA, or a control shRNA plasmid (sc-108060, Santa Cruz) encoding a scrambled shRNA sequence. These shRNAs were transfected into Huh7 and SK-HEP1 cells using Lipofectamine 2000 (Invitrogen) in the absence of serum according to the manufacture’s recommendations by addgene. After 48 h of transfection, cells stably expressing shRNA were enriched via puromycin (sc-108071B, Santa Cruz) selection. Then the medium was replaced with DMEM, and cells were cultured for another 24 h. Finally, the transfected cells were harvested to analyze the efficiency of Chibby knockdown.

### 4.9. Colonies Formation Assay

After transfection for 24 h, cells were plated into 6-well plates (1000 cells/well) and cultured in normal cultured media at 37 °C and 5% CO_2_ for 2 weeks. Then cells were fixed with 4% paraformaldehyde for 10 min and stained with 0.5% crystal violet for 30 min. The numbers of cells were quantified by image analysis using PhotoImpact (Adobe, San Jose, CA, USA). Each group was represented in the form of triplicates.

### 4.10. Invasion Assay

Cell invasion assays were performed on Boyden chamber with 8-μm pore-size filter with a basement membrane matrix (BD Bioscience Matrigel, San Jose, CA, USA). Briefly, cells were trypsinized and resuspended in serum-free media and seeded onto 8-μm polycarbonate membranes coated with a basement membrane matrix. After 4 h incubation, cells migrated through the pores, and the cells attached to the underside of the membrane were stained with Giemsa. Finally, the number of cells was calculated under the microscope. Each group was represented in the form of triplicates.

### 4.11. Statistical Analyses

Experimental values of continuous variables are expressed as the mean ± standard error of the mean. Two-tailed, unpaired Student’s *t*-test was statistically evaluated to compare the differences between the groups. Curves for recurrence and survival rates were determined by the Kaplan–Meier analysis and compared using the log-rank test. Cox’s proportional hazards model was used to determine the independent factors of recurrence and survival based on the variables selected in the univariate analysis. Statistical analysis was performed with the SPSS software package for Windows (SPSS 22.0 for Windows; SPSS Inc., Chicago, IL, USA). All *p*-values were two-tailed, and a *p*-value of less than 0.05 was considered to be statistically significant.

## 5. Conclusions

In conclusion, the study we present here has indicated the high nuclear expression of Chibby predicted good prognosis in HCC patients with high nuclear expression of β-catenin compared with those with low nuclear expression of Chibby, which implies Chibby plays a salvage role in HCC with β-catenin activation. The expression of Chibby and β-catenin in nuclear may be useful as novel prognostic predictors and therapeutic targets for HCC patients.

## Figures and Tables

**Table 1:** Correlation of nuclear expression of β-catenin/Chibby with clinicopathological characteristics in 156 patients with hepatocellular carcinoma (HCC).

Clinicopathologic	β-Catenin (high)/Chibby (high) (n = 13)	β-Catenin (high)/Chibby (low) (n = 18)	β-Catenin (low)/Chibby (high) (n = 59)	β-Catenin (low)/Chibby (low) (n = 66)	*p* value
Age (>60 vs. ≤60 years)	4/9	9/9	15/44	28/38	0.125
Sex (Male vs. Female)	11/2	13/5	46/13	55/11	0.689
AFP (>200 vs. ≤200 ng/L)	6/7	8/10	26/33	25/41	0.875
HBsAg (positive vs. negative)	9/4	12/6	40/16	47/16	0.914
HCV Ab (positive vs. negative)	3/10	3/15	14/42	15/48	0.909
Liver cirrhosis (yes vs. no)	7/6	11/7	38/21	78/28	0.837
Tumor size (>5 vs. ≤5 cm)	10/3	11/7	30/29	32/34	0.250
Tumor number (multiple vs. single) *	2/11	9/9	16/43	11/55	0.025
TNM Stage (III + IV vs. I + II)	9/4	13/5	28/31	30/36	0.108
Histology grade (Poor vs. Well + Moderate)	6/7	6/12	17/42	15/51	0.349

HBsAg: hepatitis B surface; HCV Ab: hepatitis C antibody; AFP: alpha-fetoprotein; The mean score of β-catenin (28.3 ± 10.3, mean SE) and Chibby (34 ± 8.7) per field (0.74 mm^2^) was calculated from 5 fields (400×magnification). We defined the “high” if the detected value more than the mean score and the “low” if the detected value less than the mean score. * β-catenin (high)/Chibby (low) vs. β-catenin (high)/Chibby (high), *p* = 0.041; β-catenin (high)/Chibby (low) vs. all the other groups, *p* = 0.007.

**Table 2:** Univariate and multivariate analysis of factors associated with HCC recurrence.

		Univariate		Multivariate	
Variable	Comparison	HR (95%CI)	*p* value	HR (95%CI)	*p* value
Age (years)	>60 vs. ≤60	0.755 (0.513–1.110)	0.153		NS
Sex	Male vs. Female	1.362 (0.856–2.167)	0.193		NS
AFP (ng/mL)	>200 vs. ≤200	1.676 (1.160–2.421)	0.006	1.907 (1.297–2.803)	0.001
HBsAg	Positive vs. Negative	1.306 (0.855–1.996)	0.217		NS
HCV Ab	Positive vs. Negative	0.811 (0.519–1.267)	0.358		NA
Liver cirrhosis	Yes vs. No	1.494 (1.001–2.169)	0.049	1.795 (1.201–2.683)	0.004
Tumor size (cm)	>5 vs. ≤5	1.904 (1.313–2.761)	0.001		NS
Tumor number	Multiple vs. Single	1.534 (1.019–2.310)	0.041		NS
TNM stages	III + IV vs. I + II	2.730 (1.870–3.983)	<0.001	2.808 (1.898–4.156)	<0.001
Histology grade	Poor vs. Well + Moderate	1.557 (1.052–2.304)	0.027		NS
β-catenin	High vs. Low	1.505 (0.971–2.332)	0.068		NS
Chibby	High vs. Low	0.802 (0.556–1.157)	0.239		NS
β-catenin/Chibby	High/Low vs. others	2.450 (1.448–4.144)	0.001	1.986 (1.160–3.400)	0.012

HBsAg: hepatitis B surface; HCV Ab: hepatitis C antibody; AFP: alpha-fetoprotein; NS: not significant; NA: not adopted.

**Table 3:** Univariate and multivariate analysis of factors associated with overall survival.

		Univariate		Multivariate	
Variable	Comparison	HR (95%CI)	*p* value	HR (95%CI)	*p* value
Age (years)	>60 vs. ≤60	0.966 (0.633–1.474)	0.892		NA
Sex	Male vs. Female	1.204 (0.719–2.014)	0.480		NA
AFP (ng/mL)	>200 vs. ≤200	1.580 (1.050–2.377)	0.028	1.638 (1.076–2.493)	0.021
HBsAg	Positive vs. Negative	1.264 (0.787–2.031)	0.333		NA
HCV Ab	Positive vs. Negative	0.804 (0.489–1.322)	0.389		NA
Liver cirrhosis	Yes vs. No	1.307 (0.854–2.001)	0.217		NS
Tumor size (cm)	>5 vs. ≤5	2.407 (1.343–3.119)	0.001		NS
Tumor number	Multiple vs. Single	1.221 (0.762–1.956)	0.407		NA
TNM stages	III + IV vs. I + II	3.351 (2.165–5.188)	<0.001	3.135 (1.979–4.968)	<0.001
Histology grade	Poor vs. well + moderate	1.858 (1.212–2.850)	0.004		NS
β-catenin	High vs. Low	1.612 (0.996–2.609)	0.052		NS
Chibby	High vs. Low	0.756 (0.501–1.140)	0.182		NS
β-catenin/Chibby	High/Low vs. others	2.534 (1.471–4.363)	0.001	2.207 (1.270–3.835)	0.005

HBsAg: hepatitis B surface; HCV Ab: hepatitis C antibody; AFP: alpha-fetoprotein; NS: not significant; NA: not adopted.

**Table 4:** Clinicopathological features of 156 patients with HCC undergoing hepatectomy.

Patient Demographics	
Age [years; median (range)]	56 (5–82)
Sex (M:F)	125:31
AFP [ng/mL; median (range)]	87 (2–80000)
Tumor size [cm; median (range)] a	5 (1–20)
Tumor number (single:multiple)	37:119
Liver cirrhosis, n (%)	94 (59.9)
Hepatitis (B:C:B+C:NBNC)	101:28:7:20
TNM stage (I:II:III:IV)	22:54:46:34
**Pathological Features**	
Capsule (Yes:No)	69:86
Microvascular invasion (Yes:No)	76:77
Histological grade (well:moderate:poor)	38:74:44

^a^ Measured by the length of the largest tumor nodule.

## Supplementary Materials

Supplementary materials can be found at https://www.mdpi.com/1422-0067/21/6/2060/s1.

## References

[1] Torre L.A., Bray F., Siegel R.L., Ferlay J., Lortet-Tieulent J., Jemal A. (2015). Global cancer statistics, 2012. CA A Cancer J. Clin..

[2] Llovet J.M., Burroughs A., Bruix J. (2003). Hepatocellular carcinoma. Lancet.

[3] Thorgeirsson S.S., Grisham J.W. (2002). Molecular pathogenesis of human hepatocellular carcinoma. Nat. Genet..

[4] De La Coste A., Romagnolo B., Billuart P., Renard C.A., Buendia M.A., Soubrane O., Fabre M., Chelly J., Beldjord C., Kahn A. (1998). Somatic mutations of the beta-catenin gene are frequent in mouse and human hepatocellular carcinomas. Proc. Natl. Acad. Sci. USA.

[5] Llovet J.M., Zucman-Rossi J., Pikarsky E., Sangro B., Schwartz M., Sherman M., Gores G. (2016). Hepatocellular carcinoma. Nat. Rev. Dis. Primers.

[6] Schulze K., Imbeaud S., Letouze E., Alexandrov L.B., Calderaro J., Rebouissou S., Couchy G., Meiller C., Shinde J., Soysouvanh F. (2015). Exome sequencing of hepatocellular carcinomas identifies new mutational signatures and potential therapeutic targets. Nat. Genet..

[7] Anastas J.N., Moon R.T. (2013). WNT signalling pathways as therapeutic targets in cancer. Nat. Rev. Cancer.

[8] Inagawa S., Itabashi M., Adachi S., Kawamoto T., Hori M., Shimazaki J., Yoshimi F., Fukao K. (2002). Expression and prognostic roles of beta-catenin in hepatocellular carcinoma: Correlation with tumor progression and postoperative survival. Clin. Cancer Res..

[9] Nhieu J.T., Renard C.A., Wei Y., Cherqui D., Zafrani E.S., Buendia M.A. (1999). Nuclear accumulation of mutated beta-catenin in hepatocellular carcinoma is associated with increased cell proliferation. Am. J. Pathol..

[10] Takemaru K., Yamaguchi S., Lee Y.S., Zhang Y., Carthew R.W., Moon R.T. (2003). Chibby, a nuclear beta-catenin-associated antagonist of the Wnt/Wingless pathway. Nature.

[11] Tolwinski N.S., Wieschaus E. (2004). A nuclear function for armadillo/beta-catenin. PLoS Biol..

[12] Shtutman M., Zhurinsky J., Simcha I., Albanese C., D’Amico M., Pestell R., Ben-Ze’ev A. (1999). The cyclin D1 gene is a target of the beta-catenin/LEF-1 pathway. Proc. Natl. Acad. Sci. USA.

[13] He T.C., Sparks A.B., Rago C., Hermeking H., Zawel L., da Costa L.T., Morin P.J., Vogelstein B., Kinzler K.W. (1998). Identification of c-MYC as a target of the APC pathway. Science.

[14] Takemaru K., Fischer V., Li F.Q. (2009). Fine-tuning of nuclear-catenin by Chibby and 14-3-3. Cell Cycle.

[15] Fischer V., Brown-Grant D.A., Li F.Q. (2012). Chibby suppresses growth of human SW480 colon adenocarcinoma cells through inhibition of beta-catenin signaling. J. Mol. Signal.

[16] Xu J., Ren G., Zhao D.A., Li B.A., Cai C.F., Zhou Y., Luo X.Y. (2014). Downregulated chibby in laryngeal squamous cell carcinoma with increased expression in laryngeal carcinoma Hep-2 cells. Oncol. Rep..

[17] Xu H.T., Li Q.C., Dai S.D., Xie X.M., Liu D.I., Wang E.H. (2011). The expression patterns and correlations of chibby, beta-catenin, and DNA methyltransferase-1 and their clinicopathological significance in lung cancers. APMIS.

[18] Collins F.S., Varmus H. (2015). A new initiative on precision medicine. N. Engl. J. Med..

[19] Bruix J., Sherman M. (2005). Management of hepatocellular carcinoma. Hepatology.

[20] Bruix J., Sherman M., Llovet J.M., Beaugrand M., Lencioni R., Burroughs A.K., Christensen E., Pagliaro L., Colombo M., Rodes J. (2001). Clinical management of hepatocellular carcinoma. Conclusions of the barcelona-2000 easl conference. european association for the study of the liver. J. Hepatol..

[21] Edge S.B., Compton C.C. (2010). The American Joint Committee on Cancer: The 7th edition of the AJCC cancer staging manual and the future of TNM. Ann. Surg. Oncol..

[22] Edmondson H.A., Steiner P.E. (1954). Primary carcinoma of the liver: A study of 100 cases among 48,900 necropsies. Cancer.

