# Supplementary material for: Combined Chibby and β-Catenin Predicts Clinical Outcomes in Patients with Hepatocellular Carcinoma
Source: Int J Mol Sci. 2020 Mar 17;21(6):2060. doi: 10.3390/ijms21062060 (PMC7139567; doi:10.3390/ijms21062060)
Supplement: Supplementary file 1 [file ijms-21-02060-s001.pdf]

Supplementary Materials

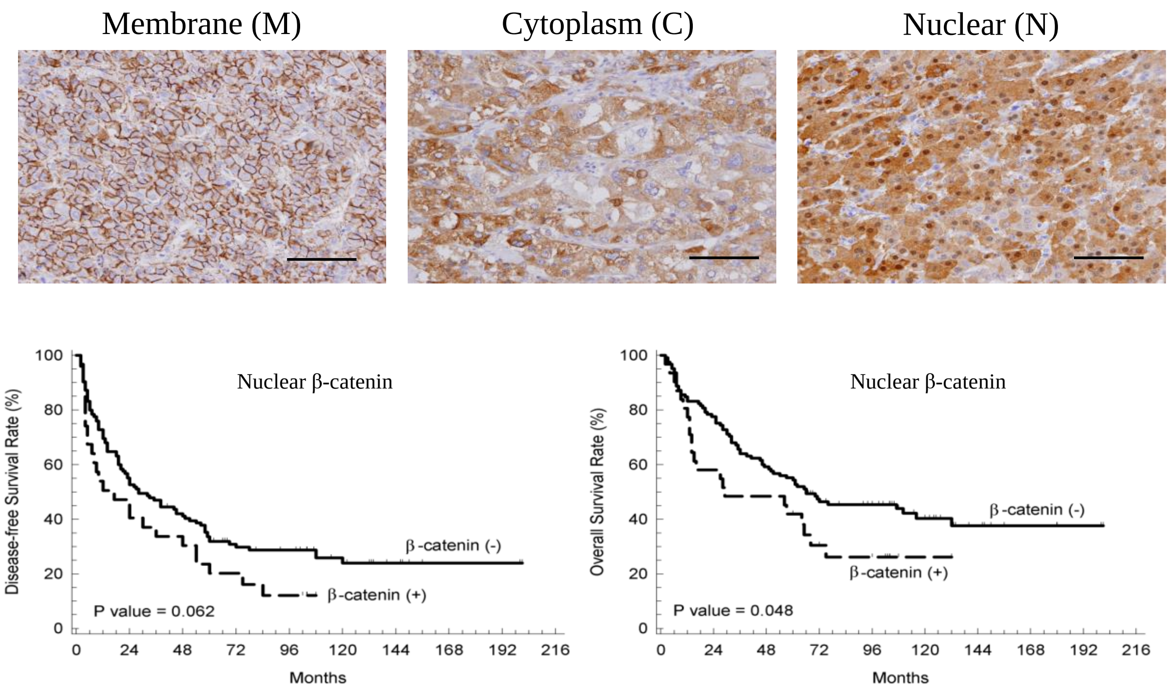

Figure S1. The IHC staining of β-catenin in 156 HCC patients. There were three types of β-catenin: membrane, cytoplasm, and nuclear. Only nuclear type had significant difference in OS (p = 0.048). Scale bars: 100 μm.

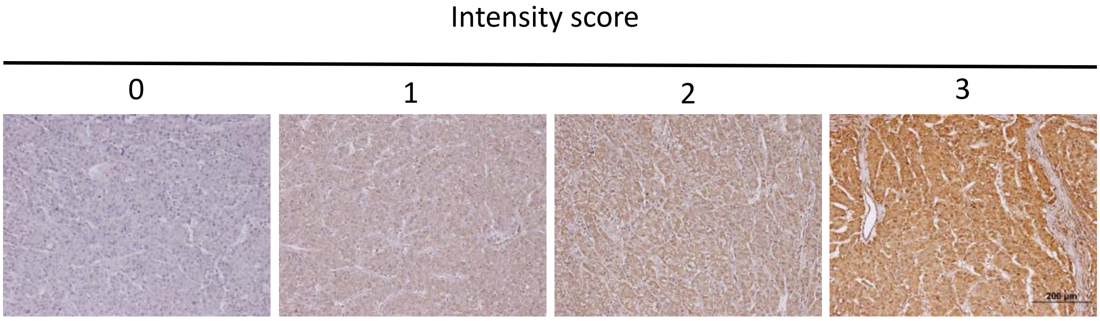

Figure S2. Intensity of immunohistochemistry staining of Chibby protein in HCC specimens [0 (null), 1 (weak), 2 (intermediate), 3 (strong)].

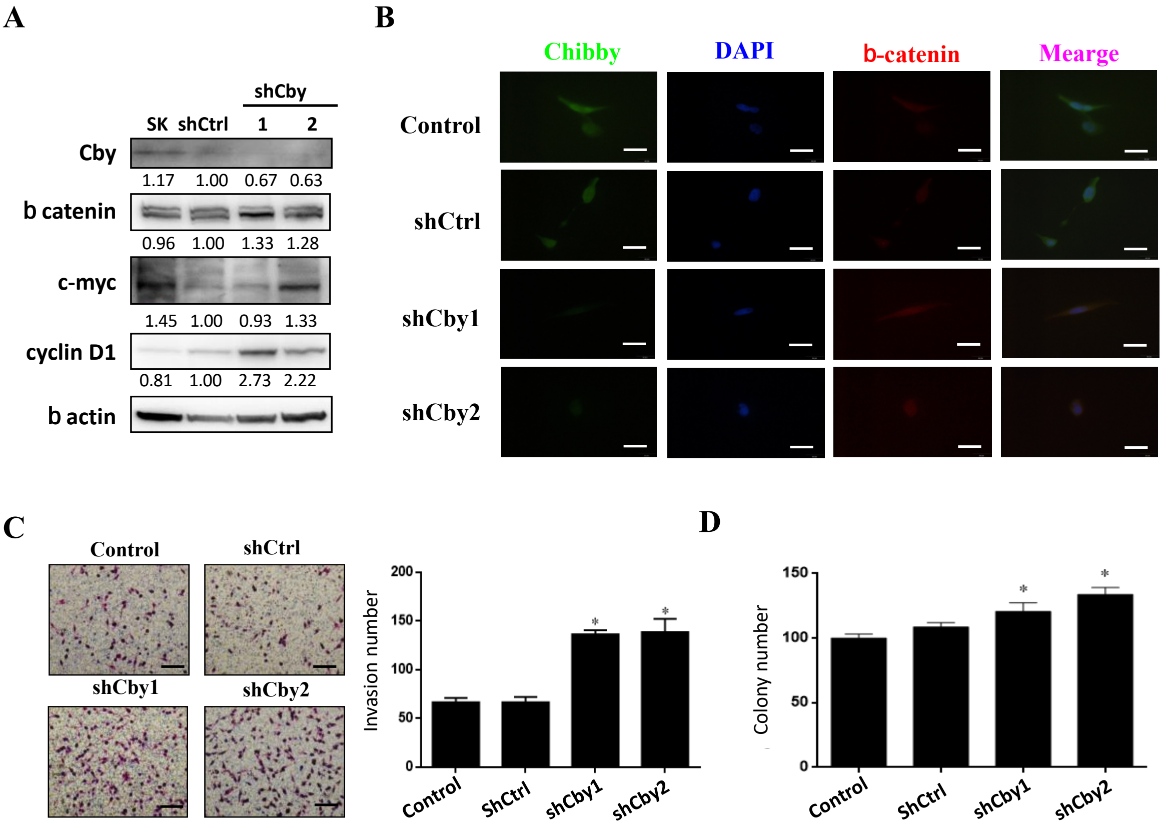

Figure S3. Knockdown of Chibby expression increases cell proliferation and invasion of HCC cells. (**A**) Two plasmids expressing *Chibby*-specific shRNA sequences were transfected into Sk-Hep-1 cells and the plasmid sh-control was designed as a scramble control. Chibby knockdown activates Wnt/β-catenin pathway. (**B**) Prominent β-catenin staining was detected in SK-Hep-1 cells treated with shChibby by immunofluorescence. Scale bars: 50 μm. (**C** & **D**) The SK-Hep-1 cells transfected with shChibby promoted cell proliferation and invasiveness by colon-formation assay and Boyden chamber system, respectively. β-actin was used as the loading control. Data represent mean ± SE from three independent analyses. Scale bars: 50 μm, * $p < 0.05$ vs. shControl.

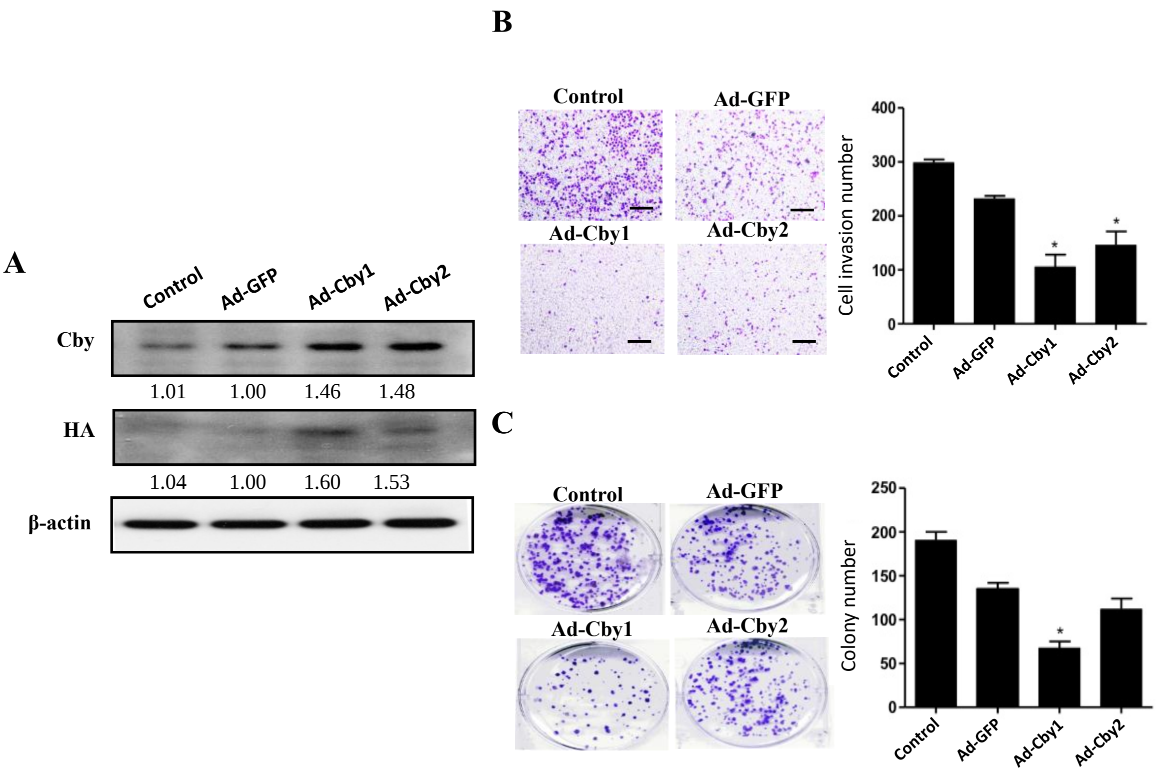

Figure S4. Overexpression of Chibby expression inhibits cell proliferation and invasion of HCC cells. (**A**) Adenovirus-mediated ectopic expression of Chibby efficiently enhanced the expression of Chibby in SK-Hep-1 cells. After 48 hours of infection with adenoviral vectors (Ad-GFP or Ad-Chibby) at different multiplicity of infection, the protein lysates from the SK-Hep-1 cells were harvested to determine the ectopic gene expression using western blot analysis. Ad-GFP was designed as the control vector. (**B** & **C**) The enhancement of Chibby in SK-Hep-1 cells transfected with Ad-Chibby suppressed cell proliferation and invasiveness by colon-formation assay and Boyden chamber system, respectively. Data represent mean ± SE from three independent analyses. Scale bars: 50 µm, * $p < 0.05$ vs. Ad-GFP.

Table S1. Summary the disease-free survival and overall survival by Chibby and β-catenin immunohistochemical staining in HCC tissue.

			**Disease-free survival**		**Overall survival**	
Protein	Location	Positive staining	HR (95% CI)	*p* value	HR (95% CI)	*p* value
Chibby	Tumor-Cytoplasm	122 (78.2%)	1.025 (0.658–1.597)	0.912	1.187 (0.710–1.987)	0.513
	Tumor-Nuclear	72 (46.2%)	1.247 (0.864–1.799)	0.239	1.324 (0.877–1.997)	0.182
β-catenin	Tumor-Membrane	96 (61.5%)	1.042 (0.716–1.516)	0.830	0.963 (0.633–1.465)	0.860
	Tumor-Cytoplasm	42 (26.9%)	1.304 (0.877–1.940)	0.190	1.054 (0.666–1.667)	0.154
	Tumor-Nuclear	31 (19.9%)	1.505 (0.971–2.332)	0.062	1.612 (0.996–2.607)	0.048
